# Exploring the prognostic significance of PKCε variants in cervical cancer

**DOI:** 10.1186/s12885-023-11236-z

**Published:** 2023-09-04

**Authors:** Sameen Zafar, Khushbukhat Khan, Yasmin Badshah, Kanza Shahid, Janeen H. Trembley, Amna Hafeez, Naeem Mahmood Ashraf, Hamid Arslan, Maria Shabbir, Tayyaba Afsar, Ali Almajwal, Suhail Razak

**Affiliations:** 1 Department of Healthcare Biotechnology, Atta-Ur-Rahman School of Applied Biosciences, National University of Sciences and Technology, Islamabad, Pakistan; 2 Department of Laboratory Medicine and Pathology, University of Minnesota, Minneapolis, MN USA; 3 Masonic Cancer Center, University of Minnesota, Minneapolis, MN USA; 4 Minneapolis VA Health Care System Research Service, Minneapolis, MN USA; 5 School of Biochemistry and Biotechnology, University of the Punjab, Lahore, Pakistan; 6 University of Bonn, LIMES Institute (AG-Netea), Carl-Troll-Str. 31, 53115 Bonn, Germany; 7 Department of Community Health Sciences, College of Applied Medical Sciences, King Saud University, Riyadh, Saudi Arabia

**Keywords:** PKCε, Cervical cancer, Genotyping, Protein-protein interactions, Molecular docking, Molecular dynamics simulations

## Abstract

**Background:**

Protein Kinase C-epsilon (PKCε) is a member of the novel subfamily of PKCs (nPKCs) that plays a role in cancer development. Studies have revealed that its elevated expression levels are associated with cervical cancer. Previously, we identified pathogenic variations in its different domains through various bioinformatics tools and molecular dynamic simulation. In the present study, the aim was to find the association of its variants rs1553369874 and rs1345511001 with cervical cancer and to determine the influence of these variants on the protein-protein interactions of PKCε, which can lead towards cancer development and poor survival rates.

**Methods:**

The association of the variants with cervical cancer and its clinicopathological features was determined through genotyping analysis. Odds ratio and relative risk along with Fisher exact test were calculated to evaluate variants significance and disease risk. Protein-protein docking was performed and docked complexes were subjected to molecular dynamics simulation to gauge the variants impact on PKCε’s molecular interactions.

**Results:**

This study revealed that genetic variants rs1553369874 and rs1345511001 were associated with cervical cancer. Smad3 interacts with PKCε and this interaction promotes cervical cancer angiogenesis; therefore, Smad3 was selected for protein-protein docking. The analysis revealed PKCε variants promoted aberrant interactions with Smad3 that might lead to the activation of oncogenic pathways. The data obtained from this study suggested the prognostic significance of PRKCE gene variants rs1553369874 and rs1345511001.

**Conclusion:**

Through further in vitro and in vivo validation, these variants can be used at the clinical level as novel prognostic markers and therapeutic targets against cervical cancer.

## Background

Cervical cancer has the fourth highest incidence rate and remains the fourth highest cause of cancer-related deaths in females around the world, with 342,000 deaths and 604,000 new cases reported globally in 2020. According to WHO cancer statistics in 2020 cervical cancer is the one of the most prevalent cancer in Pakistani female population with third highest mortality rate and 3,197 estimated deaths [1, 2]. Chronic infections with high-risk human papillomavirus (HPV) are responsible for 99.7% of cases of cervical cancer [3]. However, progression to cervical cancer is a gradual process and involves three stages of pre-cancer lesions (i.e., cervical intraepithelial neoplasia: CIN-I, CIN-II, CIN-III) prior to the development of invasive cervical cancer [4, 5]. Factors other than HPV infection, including reproductive and sexual factors, oral contraceptives, family history, tobacco smoking, and socioeconomic status. Moreover, various studies have proved that genetic factors of hosts such as SNPs, also contribute to the development of cervical lesions and cervical cancer [6, 7].

PKCs belong to AGC-kinase family of serine/threonine kinases, and they are known to act as intersection points in various cellular processes and signal-transduction pathways [8, 9]. PKCε (alternative name KPCE) is an isoform in the calcium-independent novel PKC sub-family [10]. It is a multi-functional protein involved in various cellular processes, encoded by PRKCE gene [11, 12]. Studies have shown that it is a transforming oncogene and its elevated levels regulate various signal transducers in oncogenic processes in various malignancies including cervical cancer [13–17]. Various studies have reported that expression of PKC isoforms is elevated in cervical cancer [18, 19].

Single nucleotide polymorphisms (SNPs) are the most common form of genetic variants occurring in human [20]. SNPs in the coding sequence of genes might have deleterious effects on the structure and function of the protein that may lead to or contribute to oncogenesis [21, 22]; as such, SNPs can be regarded as the genetic basis and as potential genetic markers for cancer susceptibility of an individual [23]. There are various SNPs identified in genes IL10, TP53, XRCC1, PAX8, CLPTM1L, HLA-A, and TGFBR2 which are associated with cervical carcinoma in different populations globally [4, 24, 25]. Genetic variants in PRKCE alter the structure and functions of the protein, ultimately leading towards cancer [26]. A few SNPs in PKC isoforms (rs454006 of PRKCG; rs546950 and rs4955720 of PRKCI; rs9907521 of PRKCA) have been reported to be associated with various cancers [27–31]. Recently, Khan et al. reported that several missense variants present in different domains of PKCε are responsible for altering the normal functioning of the protein and might play roles in disease contribution. The C2-like regulatory domain of PKCε is involved in regulating interactions with proteins, which are essential for controlling PKCε activation. Variants present in the regulatory domain tend to affect PKCε`s flexibility, leading to deviation in the ability of PKCε to make protein-protein interactions that might be associated with disease progression [32]. In a recent study it was indicated that the PRKCE variant rs1553369874 was associated with HCV-induced HCC [33]. However, so far, no published data has reported the association of these variants with the risk of cervical cancer in in vivo or in vitro studies.

In this study, two missense variants rs1553369874 and rs1345511001 of PRKCE, resulting in amino acid substitutions E14K and D39H, respectively, in the C2-like regulatory domain of the protein were selected. Their genotype frequencies were determined in cervical cancer patients and healthy control groups to reveal their possible association with the risk of cervical cancer in Pakistani population. Additionally, the findings of this study can be extended beyond the Pakistani population to the global context in the future, as these PKCε variants might be associated with the risk of cervical cancer in other populations as well, hence holding the potential to be used as a novel biomarker for the prognosis of cervical cancer.

Protein interactions studies have proven to be helpful in the identification of cancer biomarkers by providing the knowledge about the physiological functioning of these biomolecules and their interactions occurring in an organism [34, 35]. Therefore, to evaluate the functional impact of these variants on the protein-protein interactions of PKCε, molecular docking was performed using HADDOCK [36] with Smad3, an essential component in TGF-β signaling pathway known to be regulated by PKCε [16, 37]. Smad3 is also known to mediate carcinogenic processes, particularly in cervical cancer [38, 39]. Lastly, the predicted docked structures were evaluated through molecular-dynamic simulations for further analysis and to attain improved insight to the molecular interactions of PKCε.

## Methodology

### Sample collection and processing

Prior to the initiation of current study, ethical approval from institutional review board (IRB No. 10-2021-01/01) was obtained from parent department Atta-ur-Rahman School of Applied Biosciences (ASAB) of National University of Sciences and Technology (see online source 1). Written and oral informed consent were attained from each participant before the collection of blood samples (see online source 2). All methods were carried out in accordance with relevant guidelines and regulations.

In this study, 95 female cervical cancer patients were ascertained from combined military hospital (CMH) after a thorough process of histological diagnosis. An equal number of control samples were also randomly collected. Patients suffering from HPV-induced cervical cancer were included for this study, while patients suffering with co-morbidity leading to cervical cancer and HIV co-infections were excluded from this study. Blood samples were obtained from the participants and stored in anticoagulating EDTA tubes for genotype analysis.

### Genotype analysis

DNA from whole blood samples of study subjects was extracted through organic (phenol-chloroform) extraction protocol [40]. The primers for variants analysis were designed using Primer 1 software [41]. Four primers were designed for genotyping of each genetic variant. Two outer primers and two internal primers were designed in such a manner that they must amplify that region of the gene which carrying the targeted variants. Primers were also validated by UCSC *In silico* PCR [42]. The genotyping analysis for variants rs1553369874 (E14K) and rs1345511001 ( D39H) was performed through Tetra-ARMS PCR in Veriti™ 96-Well Thermal Cycler of Applied Biosystems™. Reaction mixture of 25 µl was prepared for every sample using Solis BioDyne FIREPol® Master Mix with 7.5 mM MgCl_2_. The primer sequences and parameters for PCR-reaction are given in Table 1. The amplified product was analyzed by agarose electrophoresis (2% W/V) under UV-transilluminator.

**Table 1:** Sequences and parameters of primers used for genotyping of rs1553369874 and rs1345511001

Variant rs IDs	Primer sequences	Tm°C	Ta°C	Product size
	CTTCTTAAGATCAAAATCTTCA			G-allele − 196
**rs1553369874(G/A)**	CTTCAAGCTCACGGCATC	53.9 ^0^ C	72 ^0^ C	A-allele − 169
	CACAAGGTGTAGGGAGTGT			Outer-band − 325
	GCTGTTGGTCTTCTGCTT			
	CGCAGACTTTCCTTCACC			G-allele − 192
**rs1345511001 (G/C)**	AGGGCAATGTAGGGCTC	55.6 ^0^ C	72 ^0^ C	C-allele − 212
	TTCTTCATTCCTGCCCTC			Outer-band − 369
	TCAAACTGGATGGTGCAG			

### Statistical analysis

Statistical analysis on obtained genotyping data was performed using GraphPad prism 9.0 software (California, USA). Fisher exact test was applied on both cervical cancer and control samples. The analysis of Odds ratios and relative risk was also performed and their respective confidence-intervals were also identified. The P-value ≤ 0.05 was regarded as statistically significant.

### Predicting the effect of variants on secondary structures of mRNA

To predict and study the effect of genetic variants on the secondary structure of PKCε mRNA an online mRNA structure prediction bioinformatics tool RNA-fold was used (http://rna.tbi.univie.ac.at/) [43, 44]. The minimum free energy (MFE) for wildtype and variants mRNA was predicted. MFE and secondary structures of wild-type and variants mRNAs were compared and analysed for the structural stability.

### Protein-protein interaction analysis

To determine the impact of amino acid variants in PKCε on its interactions with target proteins, molecular docking was performed. The 3D structure of Smad3 used in molecular docking simulation was predicted from I-TASSER https://zhanglab.ccmb.med.umich.edu/I-TASSER/ that use fold recognition/threading approach for 3D modeling of protein structures [45]. 3D structure of Smad3 was selected based on the highest C-score value depicting the global accuracy of the predicted model as well as low RMSD and local error values. The protein structures were further validated using Ramachandran analysis using PROCHECK [46]. Smad3 was chosen after the verification from literature that it is a target protein of PKCε and it is involved in the regulation of cancer cell metabolism and proliferation [16, 37, 47]. The variant structures of PKCε were attained through *In silico* mutagenesis using PyMol v4.0.4 [48]. For first variant glutamic acid at residue position 14 was substituted with lysine and aspartic acid at residue 39 was mutated to histidine in the second variant structure and they were subsequently used for molecular docking and molecular-dynamic simulations. Three Molecular docking simulations (WT-Smad3, E14K-Smad3, D39H-Smad3) were performed through HADDOCK SERVER 2.4 [36]. The cluster with lowest Z-value was selected from each docking simulation and docked structure from each cluster was chosen for further analysis.

### Interaction dynamic analysis

The dynamics of molecular interactions between native PKCε and its variants with Smad3 were studied through MD simulations using GROMACS 2016 [49], with OPLS-AA force field [50] that was used for the simulation of wildtype and variants PKCε/Smad3 complexes. A cubic box was formed around each complex for solvation by adding SPC216 water molecules, and subsequent neutralization by incorporating Na^+^ / Cl^−^ ions. Initial energy minimization of MD-simulations was performed for 50,000 steps, followed by NVT and NPT equilibration for 100ps, through steepest descent. From the same random seed, trajectories of MD complexes were initiated. MD-simulations for wildtype and variants complexes were performed for the production run of 10ns. Dynamic trajectories were constructed using in-built programs of GROMACS 2016 (gmx_ trjconv). The visualization of docked structure was performed through LIGPLOT [51] and contact analysis of MD simulations was mostly performed using VMD [52]. Several structural analyses of complexes were also performed. Calculation of root mean square deviation (RMSD for protein backbone) was performed using command gmx_ rms, radius of gyration (Rg for protein, backbone) was calculating using gmx_ gyrate command, analysis of solvent accessibility surface area (SASA) number of hydrogen bonds was performed using gmx_ sasa and gmx_ hbond commands respectively. All MD analyses were plotted as scatter line plots.

### Pathway construction

In order to construct a molecular pathway illustrating the impact of amino acid variations in PKCε and its subsequent effect on cellular signalling, Kyoto Encyclopedia of Genes and Genomes (KEGG: hsa:351) [53] and GeneMANIA [54] were employed. Furthermore, STRING database [55] was used to predict and analyze genetic interactions while functional annotation of genes and the genetic pathway was attained through DAVID database [56].

## Results

### Clinical features of cervical cancer in patients

The current study involved 95 cervical cancer patients. The general features of the patients are presented in Table 2. The ages of cervical cancer patients range from 35 years to 66 years, whereas their median age was 51 years. Furthermore, the median age of the control group was 41 years, with the range of ages from 30 years to 64 years.

**Table 2:** Clinical features of cervical cancer patients and control

Clinical characteristics		Cervical cancer	Control
**Age**	**≤** 50 years	47	81
	**>** 50 years	48	14
**Clinical stage**	Stage I-II	50	
	Stage III-IV	45	
**Metastasis stage**	Metastatic	35	
	Non-Metastatic	60	

### Association of prkce genetic variants with cervical cancer and control

DNA extracted from the samples was genotyped for the presence of missense variants rs1553369874 (g. 45,652,140 G > A) and rs1345511001 (g. 45652215G > C) in the PRKCE gene; these variants result in the substitution of Glutamic acid (E) to Lysine (K) at residue 14 and Aspartic acid (D) to Histidine (H) at position 39. Tetra ARMS-PCR was used for the process of genotyping. This technique utilizes four primers that amplify the targeted gene sequence and result in an outer control band and inner genotype bands.

The frequency distributions for genotypes of both PKCε genetic variants rs1553369874, and rs1345511001 for control and cervical cancer samples are presented in Table 3. It was found that for variant rs1553369874 genotype GG was associated with elevated risk for cervical cancer (Odds Ratio (OR) = 2.571, Relative Risk (RR) = 1.609, P = 0.0022); whereas, genotype AA was found to have protective role in this regard (OR = 0.1566, RR = 0.3080, P < 0.0001). For variant rs1345511001, genotype GG was found to be significanty (associated with increased risk of cervical cancer (OR = 2.363, RR = 1.548, P < 0.0056); whereas, genotype CC was statistically associated with a protective role in cervical cancer (OR = 0.5128, RR = 0.7031, P = 0.0456).

**Table 3:** Genotype and allele distribution and association of PRKCE variants rs1553369874 and rs1345511001 with cervical cancer

SNP	Genotype	Frequency of distribution		OR		RR		P-value
		Control	Patients	OR	95% CI	RR	95% CI	
**rs1553369874**	GG	38 40.00%	60 63.16%	2.571	1.401–4.677	1.609	1.197–2.204	0.002
	AA	32 33.68%	7 7.37%	0.1566	0.06131–0.3646	0.3080	0.1523–0.5708	< 0.0001
	GA	25 26.32%	28 29.47%	1.170	0.6229 to 2.227	1.080	0.7786–1.442	0.7465
	G	50 52.63%	74 77.89%	0.3153	0.1730–0.5988	0.5914	0.4509–0.7780	0.0004
	A	45 47.37%	21 22.11%	3.171	1.670–5.781	1.691	1.285–2.218	0.0004
**rs1345511001**	GG	41 43.16%	61 64.21%	2.363	1.327–4.281	1.548	1.149–2.127	0.0056
	CC	39 41.05%	25 26.32%	0.5128	0.2788–0.9541	0.7031	0.4897–0.9713	0.0456
	GC	15 15.79%	9 9.47%	0.5581	0.2345–1.335	0.7238	0.4018–1.142	0.2747
	G	48 50.53%	66 69.47%	0.4487	0.2547–0.8036	0.6809	0.5146–0.9016	0.0116
	C	47 49.47%	29 30.53%	2.228	1.244–3.927	1.469	1.109–1.943	0.0116

Allele frequencies of both PKCε variants are described in Table 3. Analysis revealed that the frequency of the G-allele in variant rs1553369874 was significantly higher in the cervical cancer group compared to the control group, (OR = 0.3153, RR = 0.5914, P = 0.0004). The A-allele was also found to be significantly different between the two groups, and the OR and RR indicated that a pathogenic role (OR = 3.171, RR = 1.691, P-value = 0.0004). Similarly, the frequency of the G-allele in variant rs1345511001 was higher in the cervical cancer group compared to the control group, and the G allele associated with a protective role (OR = 0.4487, RR = 0.6809, P-value = 0.0116). The frequency of the C-allele in this variant was increased in the control samples and has role in elevated risk of cervical cancer (OR = 2.228, RR = 1.469, P-value = 0.01).

### Association of prkce genetic variants with clinical characteristics of cervical cancer patients

Frequency distribution of genotypes for both variants in different age groups are given in Table 4. Genotypes GG and GA of variant rs1553369874 were not statistically significant in either age group; however, genotype AA of the variant showed statistical significance for subjects of both age groups (≤ 50 years: OR = 3.596, RR = 1.439, P = 0.0118; >50 years: OR = 26.11, RR = 5.185, P = 0.0016). The CC-genotype of variant rs1345511001 showed significance for the age group of ≤ 50 years (OR = 2.368, RR = 1.334, P = 0.0368). All other genotypes of this variant were non-significant in both age groups.

**Table 4:** Genotype distribution and association of PRKCE variants rs1553369874 and rs1345511001 with cervical cancer in age groups of ≤ 50 and > 50

SNP	Genotype	Frequency of distribution		OR		RR		P-value
		Control	Patients	OR	95% CI	RR	95% CI	
**rs1553369874**	GG (≤ 50 YEARS)	32 39.51%	26 55.32%	0.5275	0.2602–1.115	0.7882	0.5867–1.031	0.0989
	GG (> 50 YEARS	6 42.86%	34 70.83%	0.3088	0.09686–0.9917	0.4125	0.1683–1.015	0.0657
	AA (≤ 50 YEARS)	27 33.33%	6 12.77%	3.596	1.339–8.788	1.439	1.103–1.816	0.0118
	AA (> 50 YEARS)	5 35.71%	1 2.08%	26.11	2.638–311.4	5.185	2.267–10.11	0.0016
	GA (≤ 50 YEARS)	22 27.16%	15 32.61%	0.7706	0.3477–1.644	0.9070	0.6453–1.198	0.5466
	GA (> 50 YEARS)	3 21.43%	13 27.08%	0.7343	0.1955–2.795	0.7841	0.2507–2.161	> 0.9999
**rs1345511001**	GG (≤ 50 YEARS)	35 43.21%	29 61.70%	0.4723	0.2265-1.000	0.7609	0.5726–0.9923	0.0662
	GG (> 50 YEARS)	6 42.86%	32 66.67%	0.3750	0.1156–1.181	0.4737	0.1921–1.168	0.1287
	CC (≤ 50 YEARS)	34 41.98%	11 23.40%	2.368	1.081–5.020	1.334	1.024–1.717	0.0368
	CC (> 50 YEARS)	5 35.71%	14 29.17%	1.349	0.4126–4.377	1.257	0.4830–3.053	0.7444
	GC (≤ 50 YEARS)	12 14.81%	7 14.89%	0.9938	0.3598–2.566	0.9977	0.6362-1.350	> 0.9999
	GC (> 50 YEARS)	3 18.75%	2 4.17%	5.308	0.9671–31.28	2.723	0.9578–5.467	0.0949

Genotype distribution frequencies among non-metastatic and metastatic patients were also determined in Table 5. In variant rs1553369874, genotypes AA and GA were shown to be statistically correlated with cervical cancer metastasis; genotype AA has a damaging role whereas genotype GA was shown to have a protective role. None of the genotypes in variant rs1345511001 showed any statistical correlation with metastasis of cervical cancer. Lastly, the distribution of genotypes among the cervical cancer patients of stage I-II and stage III-IV were determined (Table 5). It has been reported here that only genotype AA of variant rs1553369874 showed significant correlation as a risk factor in patients with stage-III or stage-IV of cervical cancer. Genotype distribution of variants with cervical cancer patient`s clinical features is given (see online source 3).

**Table 5:** Genotype distribution and association of PRKCE variants rs1553369874 and rs1345511001 with secondary site metastatic and stages of cervical cancer

SNP	Genotype	Frequency distribution		OR	RR	P-Value	Frequency distribution		OR	RR	P-Value
		Metastatic	Non-metastatic				Stage I-II	Stage III-IV			
**rs1553369874**	GG	24 68.57%	36 60.00%	1.455	1.273	0.5095	31 62.00%	29 64.44%	0.9002	0.9518	0.8342
	AA	6 17.14%	1 1.67%	12.21	2.601	0.0094	1 2.00%	6 13.33%	7.538	1.934	0.0500
	GA	5 14.29%	23 38.33%	0.2681	0.3988	0.0189	18 36.00%	10 22.22%	1.969	1.346	0.1784
**rs1345511001**	GG	20 57.14%	41 68.33%	0.6179	0.7432	0.3750	36 72.00%	25 55.56%	2.057	1.433	0.1334
	CC	11 31.43%	14 23.33%	1.506	1.283	0.4704	11 22.00%	14 31.11%	0.6245	0.7897	0.3566
	GC	4 11.43%	5 8.33%	1.419	1.233	0.7211	3 6.00%	6 13.33%	0.4149	0.6099	0.3000

### Impact of genetic variants rs1553369874 and rs1345511001 on prkce mRNA structure 

**Fig. 1:**
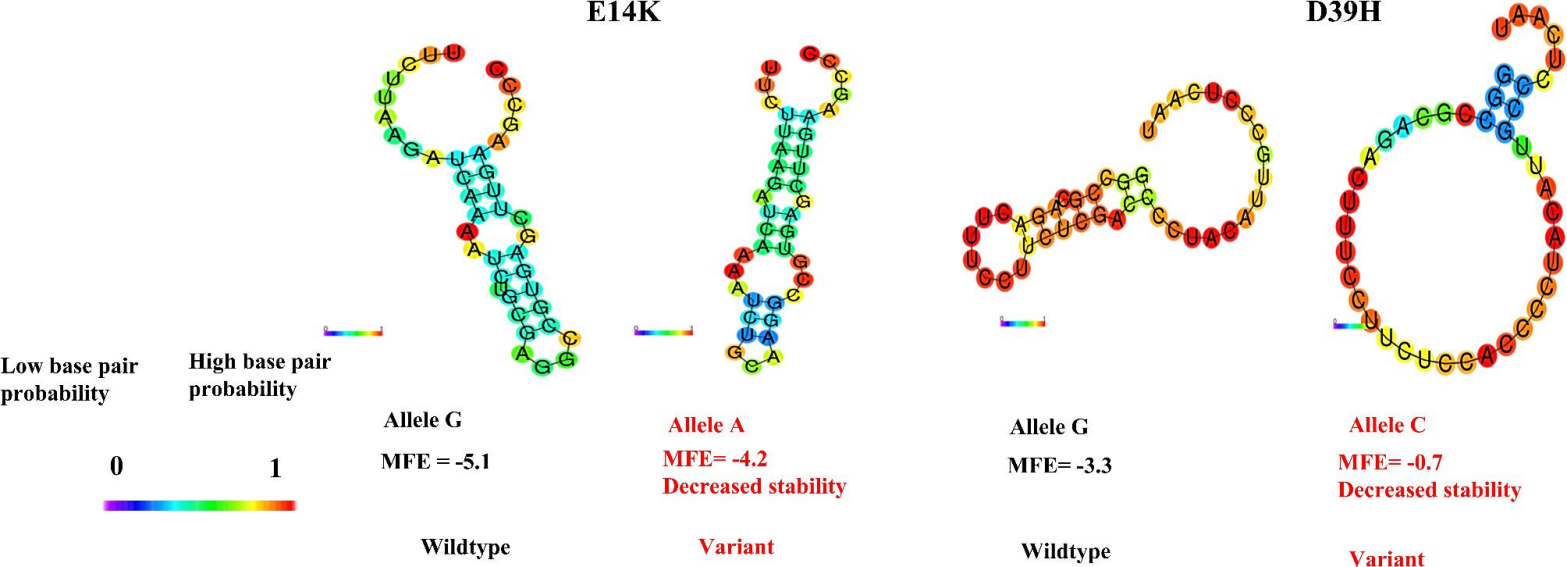
Predicted structures of mRNAs using RNAfold **(a)** rs1553369874 effect on mRNA structure due to the substitution of reference allele G (left) with variant allele A (right) **(b)** rs1345511001 effect on mRNA structure due to the substitution of reference allele G (left) with variant allele A (right) Darker colors represents high probability for base-pairing whereas lighter colors indicates that these sequences not form base-pair at their alignment position

*In silico* analysis was performed to predict the secondary structures of mRNAs of wildtype PKCε and its selected variants. Minimal free energy (MFE) for the wildtype and variants was also determined and analyzed. The secondary structures of mRNA for rs1553369874 and rs1345511001 showed a dramatic change when compared to their wildtype counterparts, demonstrating the significant effect of variant alleles on the overall structure of mRNAs (Fig. 1). MFE calculations for alleles of the variant rs1553369874 predicted that MFE for the reference G allele was − 5.1 Kcal/mol, whereas MFE was elevated for variant allele A with the value of -4.2 Kcal/mol. The variant rs1345511001 showed a decrease in MFE value for the wildtype allele G (MFE= -3.3 Kcal/mol) compared to the variant allele C with MFE value − 0.7 Kcal/mol (see online source 4). The decreased values of MFE for the reference alleles in both variants depicts increased structural stability [57]. Whereas the altered alleles for both variants had increased MFE values compared to their wildtype counterparts and thus were structurally less stable. The altered alleles in both variants are associated with cervical cancer risk (see Table 3) which might be the result of decreased mRNA structural stability.

### Analysing intramolecular interactions between PKCε and Smad3 complexes

**Fig. 2:**
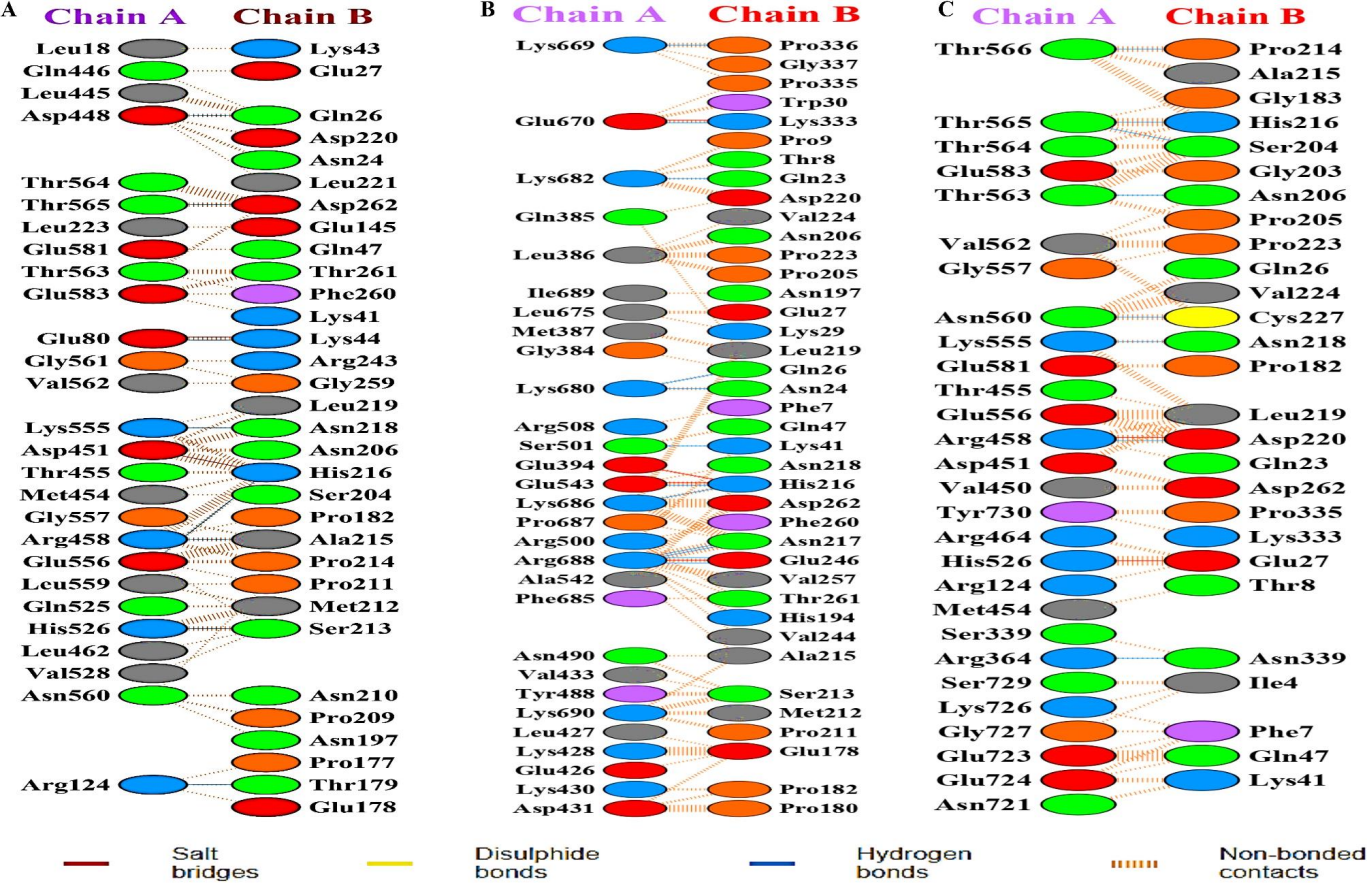
PDBsum illustration depicting list of interacting amino acid residues of chain A representing PKCε and chain B representing Smad3 **(a)** List of interacting residues in wildtype PKCε-Smad3 complex **(b)** List of interacting residues in E14K-Smad3 complex (c) List of interacting residues in D39H-Smad3 complex

Molecular interactions of PKCε with Smad3 were also determined and molecular docking was performed between PKCε (wildtype, E14K, D39H) and Smad3 to predict and compare the nature of interactions of PKCε prior to and after the occurrence of missense variations. 3-dimensional structures of Smad3 and PKCε were predicted through I-TASSER and the models with highest C-score values were selected which indicated the quality of the predicted 3D models. Three docking simulations were carried out between wildtype PKCε and Smad3, E14K and Smad3, and D39H and Smad3 using HADDOCK SERVER 2.4 and clusters of docked models were generated. The cluster with lowest value of Z-score was selected from each docking simulation, which indicates that those clusters have lowest values of standard deviation, and the best protein interaction model from each cluster was selected that corresponds to the highest score structure [58, 59]. It was particularly noteworthy that in the computational structures obtained from docking, PKCε (wildtype, E14K, D39H) approached Smad3 mostly through its catalytic domain. This finding is exactly in accordance with the previous findings that Smad3 is a phosphorylation substrate of PKCε [16, 37]. While analyzing the intramolecular interactions between native PKCε and its variants with Smad3, it was determined that variants E14K and D39H interacted with Smad3 via catalytic domain residues by forming increased numbers of hydrogen bonds and hydrophobic interactions. Whereas native PKCε interacts with Smad3 by making fewer hydrogen bonds and hydrophobic interactions. It was also observed that the wildtype PKCε and its variants are involved in strong interactions with the following residues of Smad3: Ser213, Pro214, Ala215, His216, Asn217, Asn218, Asp220. The visualization of intermolecular interactions between wildtype and variants PKCε with Smad3 in the docked structures was performed through LigPlot + v.2.2.4 as shown in online source 5. To further validate the molecular interactions of the protein-protein interaction complexes, PDBsum server was used which presents the interaction-statistics as well as depicts the protein interacting sites and interacting residues [60]. Furthermore, this tool also provides the information about the types of the molecular interactions present in protein-protein interaction complexes which includes hydrogen binds, salt-bridges, disulfide bonds, and non-bonded interactions. It was revealed that the linker domain residues of Smad3 made interactions via salt bridges, hydrogen-bonds, and non-bonding interactions with the kinase domain of PKCε that resulted in Smad3 activation **(**Fig. 2**)**.

### Interactions dynamic analysis of wildtype and variant PKCε - Smad3 complexes

In order to understand the consequences of amino acid variants on the structure of PKCε and dynamics of its interactions with Smad3, MD-simulations were performed that predict the molecular motion and behavior of protein complexes in four dimensions [61]. GROMACS tool was used to carry out simulations between wildtype PKCε and its variants with Smad3 for a time period of 10 ns and data for structural analysis of all three complexes was obtained (see online source 6).

**Fig. 3:**
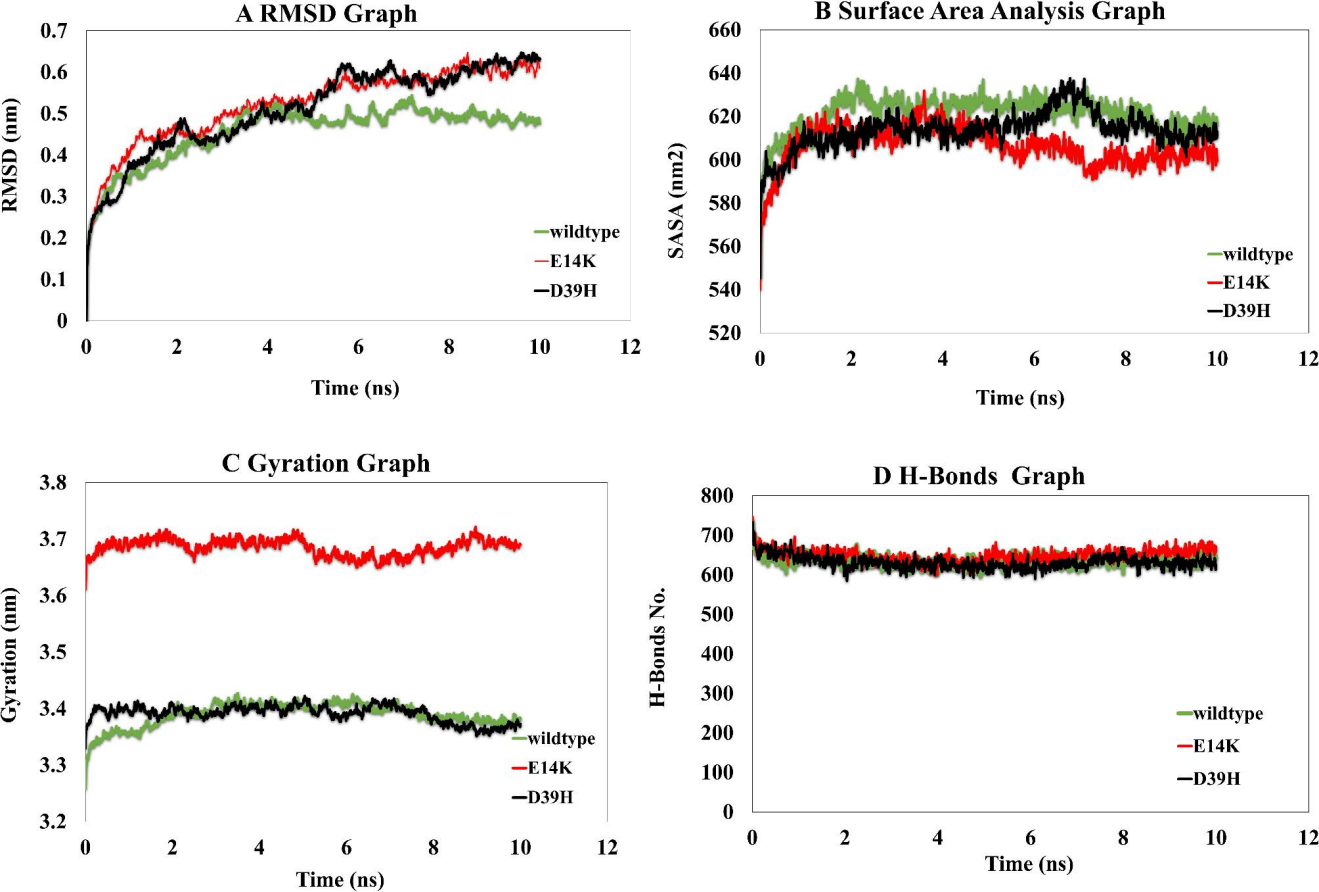
Molecular dynamic analysis of wildtype and variants PKCε-Smad3 **(a)** Root mean square deviation (RMSD) **(b)** Solvent accessibility surface area (SASA) **(c)** Radius of gyration (Rg) **(d)** Number of Hydrogen bonds

RMSD (root mean square deviation) analysis was performed to analyze the average residual deviations in the protein structures over the course of simulation [58]. RMSD values for PKCε and its variants are given in the figure (Fig. 3a). RMSD values increased to 0.3 nm for all three structures during the 0 ns – 1 ns period of simulation. This value was further increased to 0.5 nm for all structures at 4 ns. After that, RMSD became more or less stable for the wildtype PKCε structure till the end of simulation. However, RMSD for both variants started to increase from the time interval of 5 ns and reached the value of 0.63 nm till the end of simulations, though during this period RMSD values for both variants remained constant.

To evaluate the variation in accessibility of surface area of the protein complex imparted by amino acid variants, SASA (solvent accessibility surface area) analysis was carried out. The data presented in Fig. 2 demonstrates that values of SASA were significantly increased from 540 nm^2^ to 620 nm^2^ during 0 ns – 1 ns of simulations (Fig. 3b). SASA values were further increased to 640 nm^2^ for the native PKCε till 2 ns; however, after that SASA values gradually decreased and at 10 ns this value was 620 nm^2^ for wildtype PKCε. SASA values for both variants showed dramatic fluctuations in their values. Particularly, during the time frame of 6 ns – 8 ns SASA for E14K was significantly reduced to 590 nm^2^, whereas SASA for D39H was recorded to be 633 nm^2^. However, during the interval of 8 ns – 10 ns SASA for all three complex remained stable around 610 nm^2^.

To determine the effect of variants on the compactness of the protein complexes, the Rg (radius of gyration) was evaluated for MD-complexes. Data from the simulations is presented (Fig. 3c). The variant E14K has Rg value of 3.6 nm at the beginning of the simulations that reached value of 3.7 nm at 2 ns. This value remained somewhat constant till 4.8 ns. However, Rg value was slightly decreased to 3.65 nm during the time frame of 5 ns – 9 ns, but by the end of simulation, its value was approximately 3.7 nm. Rg for wildtype has initial value of 3.25 nm whereas for D39H its value is 3.35 nm. During 0 ns -2 ns, Rg for D39H remained higher compared to the wildtype but this value was approximately equal to 3.4 nm during the time period of 2 ns – 8 ns; however, its value decreased once more to approximately 3.36 nm during the last 2 ns.

Hydrogen bonds plays a critical role in the molecular interactions and maintenance of 3D structures of proteins. Number of hydrogen bonds that are formed in protein structures during the course of simulations are determined to analyze the effect of variants on protein interactions. It can be seen that during the period of 0 ns – 2 ns, the number of hydrogen bonds decreased from 700 to 600, but after that interval the number of hydrogen bonds remained stable throughout the course of simulations (Fig. 3d). Interactions dynamic analysis that the number of hydrogen bonds formed in E14K and D39H complexes are slightly higher compared to that in the wildtype PKCε, showing that the molecular interactions which are formed between variants with Smad3 are more potent and stable compared to that of the wildtype.

**Fig. 4:**
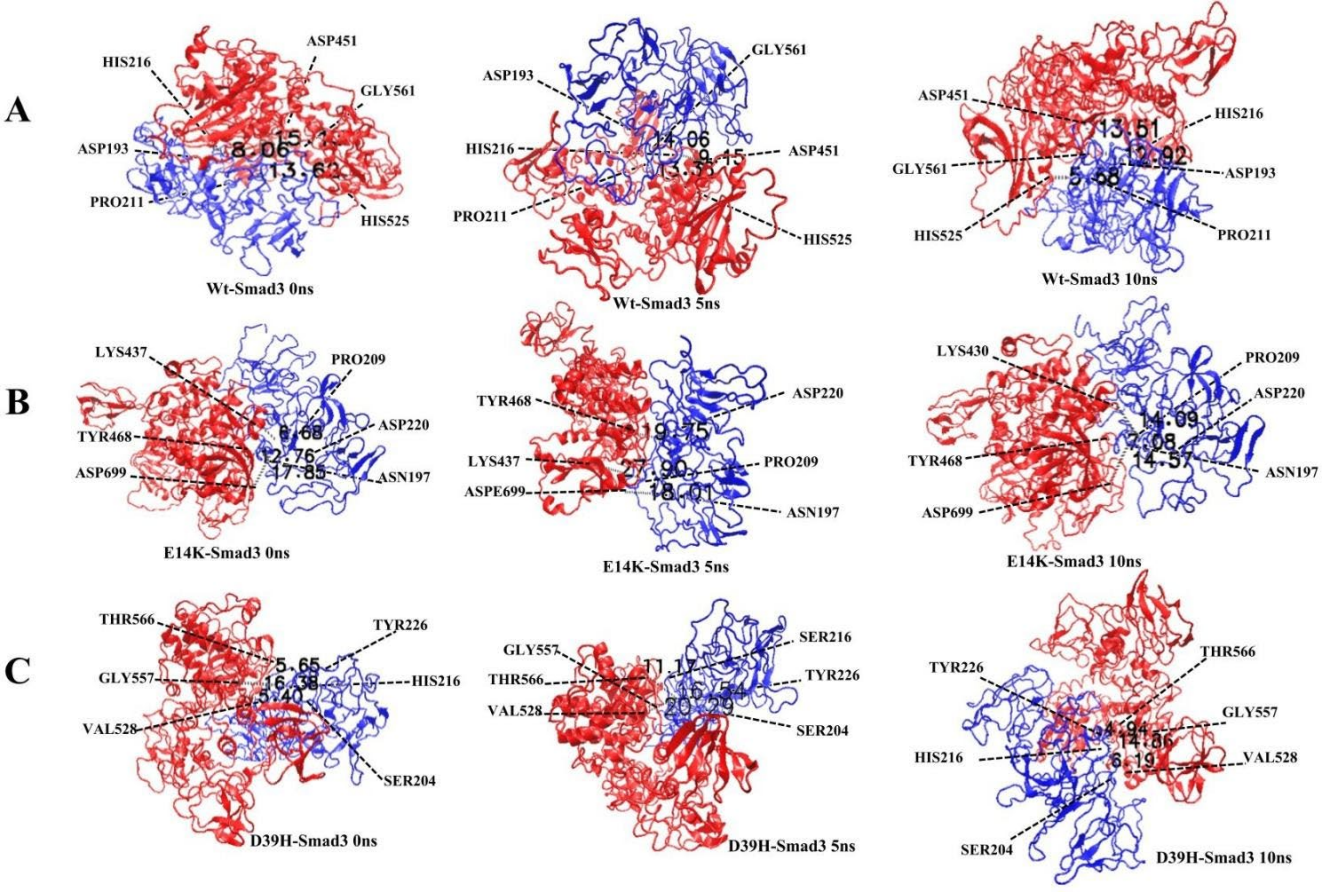
Molecular distances between PKCε and Smad3 at three random pairs of interacting residues. Red colour represents PKCε/variants protein and Blue colour represents Smad3 protein in PKCε-Smad3 complex **(a)** Molecular distances recorded for wildtype PKCε-Smad3 complexes at 0ns, 5ns, 10ns **(b)** Molecular distances recorded for E14K-Smad3 at 0ns, 5ns, 10ns **(c)** Molecular distances recorded for D39H-Smad3 at 0ns, 5ns, 10ns

The distances between interacting residues of PKCε (wildtype, E14K, D39H) and residues of Smad3 were also determined. The residues selected for this purpose were involved in intramolecular interactions by forming hydrogen bonds. Three pairs of such interacting residues were randomly selected and their distances were measured at three different time intervals (0 ns, 5 ns, 10 ns) of MD simulations. In the wildtype PKCε -Smad3 complex, the distance between the interacting residues were determined at 0 ns and 10 ns. It was shown that at 10ns the distance between Asp451 of PKCε and His216 of Smad3 was decreased to 13.51Å which was 15.14Å at 0ns. The distance between His525 of PKCε and Pro211 of Smad3 was also significantly decreased to 5.68Å at the end of simulations, which was 13.63Å at 0ns. While the distance between Gly561 and Asp193 was increased to 12.92Å from 8.06Å at the end of simulations (Fig. 4a). Similarly, distances between the interacting residues of E14K-Smad3 depicted that the bond length between the residues Asp699 (PKCε) and Asn197 (Smad3) as well as Tyr468 (PKCε) and Asp220 (Smad3) were decreased to 14.57Å and 7.08Å at 10ns compared to 17.85Å and 12.76Å at 0ns, respectively. However, the bond length between the interacting pair Lys437 (PKCε) and Pro209 (Smad3) was increased to 14.09Å from 6.68Å at the end of simulations (Fig. 4b). Lastly, bond distances between the interacting residues of D39H and Smad3 were also determined which also exhibited similar trends in their bond lengths. The distance between interacting resides Thr566 (PKCε) and Tyr226 (Smad3) was 4.94Å at 10ns, while at 0ns the bond distance was recorded to be 5.65Å. Similarly, the bond distance between Gly557 (PKCε) and His216 (Smad3) was reduced to 14.86Å at 10ns from 16.38Å recorded at the start of the MD simulations (Fig. 4c). Distances between the interacting residue pairs for all three complexes are described in Table 6.

**Table 6:** Bond distance between PKCε and Smad3 interacting residues

Complex	Residue pair 1	Distance	Residue pair 2	Distance	Residue pair 3	Distance
**Wt.PKCε-Smad3**	Asp451(PKCε) - His216(Smad3)	15.14 A^O^ (0ns)	Gly561(PKCε) - Asp193(Smad3)	8.06 A^O^ (0ns)	His525(PKCε) - Pro211(Smad3)	13.63 A^O^ (0ns)
		9.15 A^O^ (5ns)		14.06 A^O^ (5ns)		13.36 A^O^ (5ns)
		13.51 A^O^ (10ns)		12.92 A^O^ (10ns)		5.69 A^O^ (10ns)
**E14K-Smad3**	Lys437(PKCε) - Pro209(Smad3)	6.68 A^O^ (0ns)	Tyr468(PKCε) - Asp220(Smad3)	12.76 A^O^	Asp699(PKCε) - Asn197(Smad3)	17.85 A^O^ (0ns)
		27.90 A^O^ (5ns)		19.75 A^O^ (5ns)		18.01 A^O^ (5ns)
		14.09 A^O^ (10ns)		7.08 A^O^ (10ns)		14.57 A^O^ (10ns)
**D39H-Smad3**	Thr566(PKCε) -Tyr226(Smad3)	5.65 A^O^ (0ns)	Gly557(PKCε) - Ser216(Smad3)	16.38 A^O^	Val528(PKCε) - Ser204(Smad3)	5.40 A^O^ (0ns)
		16.57 A^O^ (5ns)		11.17 A^O^ (5ns)		20.29 A^O^ (5ns)
		4.94 A^O^ (10ns)		14.86 A^O^ (10ns)		6.19 A^O^ (10ns)

## Discussion

Several studies have demonstrated that genetic variants in PKCε and its isoforms are associated with several diseases, particularly cancers [62–65] [66]. The present study has explored the role of missense variants in PRKCE in association with cervical cancer in Pakistani female population. Studies have demonstrated that PRKCE non-synonymous variants rs1553369874 and rs1345511001 might have a damaging role leading to cancer susceptibility [32]. The results of this study have suggested that variants rs1553369874 and rs1345511001 in PRKCE are associated with risk of cervical cancer susceptibility in Pakistani population.

It was suggested from the tetra ARMS-PCR data that genotypes GG of rs1553369874 and GG of rs1345511001 were associated with increased risk of cervical cancer, whereas their counterpart genotypes AA and CC were most prevalent in healthy controls and imparted protective effect in Pakistani female population. This result was consistent with reported studies where variant genotypes were not associated with disease progression, rather they were imparting a protective effect in this regard [67–70]. Though allele A of rs1553369874 and allele C of rs1345511001 had high distribution frequency in control samples, their ORs, and RRs were higher compared to that of reference alleles of both variants, respectively. This discrepancy in data might have occurred because of small cohort size.

The cervical cancer incidence rates are highest in woman of age 35–50 years, whereas they are low for females of age 51–65 years [71]. It was found that genotype AA of rs1553369874 was acting as a risk factor in women of both age groups, while only genotype CC for the variant rs1345511001 was found to have detrimental role in females of age ≤ 50 years. Association of genetic variants with tumor metastasis state and tumor stage was also determined and genotype AA of rs1553369874 was shown to be correlated with advanced stage cervical cancer and secondary site metastasis.

The findings of this research have the potential to be implicated in cervical cancer studies beyond the studied population. These PRKCE variants may contribute towards the risk of cervical cancer in various population, as similar SNPs have been corelated with the risk of cancers in previous studies. For instance, previously studies have identified genetic variants rs546950, rs4955720 in PRKCI gene which are associated with the risk of prostate cancer in Han Chinese [30] and Iranian populations [67]. Similarly variant rs1801270 in CDKN1A gene are associated with development of cervical cancer in Han Chinese [69], Brazilian [72], and Iranian populations [73]. Nonetheless, further research is required in diverse population with large cohort size to validate the finding of this study as well as to assess the global significance of these variants with cervical cancer.

Secondary structures of mRNAs of both variants and their corresponding wildtype were predicted and the difference of mRNA structures and MFE values between wildtype and variants revealed that the structural stability of wildtype mRNA was higher compared to the variant mRNAs, which could lead to the onset of diseases [57, 74]. This analysis could be verified from the fact that altered alleles A and C of variants rs1553369874 and rs1345511001 were associated with increased cervical cancer risk. Furthermore, secondary structures of mRNAs are responsible for the modulation of translation rates and any changes in mRNA structures can lead to protein misfolding and structural instability [75–77]. that can lead to severe illnesses including cancer.

Such variants that cause protein structure instability can lead to cancer progression. Destabilizing variants in E-cadherin have been reported to be associated with the development of gastric cancer at early ages compared to the variants that do not alter the protein stability [78]. Similarly, missense variants in c-src destabilized the structure of the protein and caused oncogenic transformation [76, 79]. Moreover, such structural destabilizations can also alter the protein-protein interactions [80]. Cervical cancer associated variants in PKCε may also effect its interactions with other proteins, leading towards oncogenic progression.

The effect of PKCε`s non-synonymous variants (E14K, D39H) on functions of the protein was studied along with the effect of amino acid variants on interactions of PKCε with Smad3 that are responsible for tumor cell growth [66] [81]. 3D structure of PKCε and Smad3 was predicted through I-TASSER that uses threading approach for prediction of protein structures [45]. *In silico* mutagenesis was performed on the predicted 3D structure of PKCε and the two variant proteins were attained. The variants E14K and D39H were shown to be pathogenic in previous studies [32] and this study evaluated their impact on PPI of PKCε with Smad3.

Several studies have reported the role of protein-protein interactions in identification of tumor biomarkers [34]. The integration of PPIs network analysis with computational bioinformatics techniques can be considered valuable for getting a deep insight into the intricated biochemical interactions occurring among the proteins present in a physiological system and which can give valuable information for designing potential prognostic biomarkers for cervical cancer [35, 82].

PKCε and its variants were than docked with Smad3 to determine the alteration in protein-protein interactions resulting from the single amino acid variation. The interaction parameters included score of desolvation, electrostatic and hydrophobic interactions, and hydrogen bonding [83] which were increased for the variants than in wild type. These results clearly indicate that interactions of E14K and D39H with Smad3 were stronger than that of wild type (online source 7).

The dynamics of molecular interactions of PKCε and its variants with Smad3 were analyzed through MD simulations. The number of hydrogen bonds that are formed within the complex over the course of MD simulations were also slightly higher for variants compared to wild type. This shows that intramolecular interactions between PKCε variants and Smad3 are stronger as hydrogen bonds plays a crucial role in stabilizing protein structure and its activity [84, 85]. The distance between interacting residues of all three complexes was also analyzed at three random sites. It was determined that those interacting residues of native PKCε/variants and Smad3 were linked through hydrogen bonds. During the course of simulations, the distance between those interacting residues showed alterations. The molecular distances for some residues were increased during the middle of simulations, however, their distances showed shrinkage at the end of simulations. Interactions between variant PKCε-Smad3 complexes were stronger due to extensive hydrogen bonding and shorter length of hydrogen bonds which are critical for the maintenance of stable protein interactions [86].

**Fig. 5:**
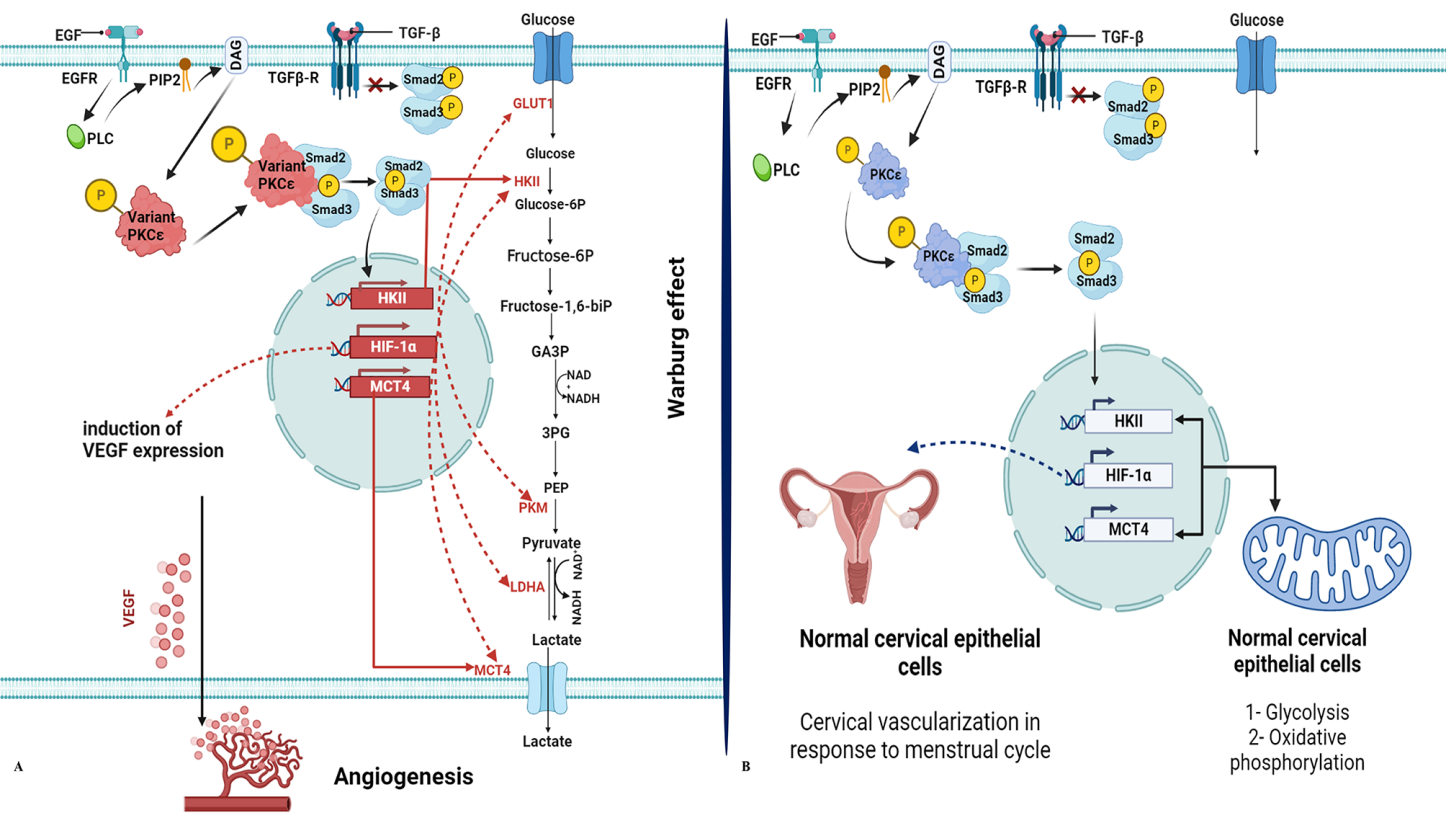
Effect of non-synonymous variants on molecular interplay between PKCε and Smad3 **(a)** Schematic illustration for crosstalk between variant PKCε and Smad3 **(b)** Schematic illustration for crosstalk between variant PKCε and Smad3

In this study analysis regarding the impact of non-synonymous PKCε variants on the crosstalk with Smad3 and the downstream molecular actors was performed. For this purpose, several genetic and protein-interactions databases were used. The information regarding gene-functional annotation and genetic linkage was attained through an extensive literature review and this information was also obtained from several databases including KEGG, STRING, geneMANIA, and DAVID. It was revealed from the studies that PKCε phosphorylates Smad3 at its linker region serine residue resulting in activation and nuclear translocation in a Tgf-β independent manner [81, 87]. Inside the nucleus activated Smad3 binds the promoter regions of several target genes and activates their transcription. It has been revealed through docking simulations and interaction dynamics analysis that variant PKCε proteins interact with Smad3 in an aberrant manner compared to their native counterpart. The binding affinity between the variant PKCε proteins and Smad3 is significantly higher compared to that in the wildtype-Smad3 complex. Since the binding interactions between both variants and Smad3 are much stronger, it will increase the binding energy of the kinase and Smad3 complex which in turn will lead towards the lowering of activation energy. This lowering in activation energy will assist in the elevated speed of the phosphorylation reaction. When the rates of Smad3 phosphorylation are increased, its activation levels will be aberrantly increased. These abnormally high levels of phosphorylated Smad3 will then mobilize to nucleus where they will frequently bind the promoter regions of target genes including HKII, MCT4, and Hif-1α, resulting in their increased rates of transcription. Elevated levels of these genes are known to regulate key oncogenic processes including Warburg’s effect. Moreover, Smad3-dependent increased activation of Hif-1α will further lead to the activation of several other oncogenes, especially VEGF which is a key activator of angiogenesis [88]. Activation of both these carcinogenic processes result in rapid proliferation of cancer cells, enhanced tumour survival and poor prognosis in patients (Fig. 5a). Contrarily, when wildtype PKCε forms a complex with Smad3, it does not bind with a high affinity and the binding energy between the native PKCε -Smad3 complex is substantially low. This will result in higher activation energy to accomplish the phosphorylation reaction. Hence, the PKCε induced activation of Smad3 will not increase aberrantly and typical levels of activated Smad3 will localize to the nucleus to activate gene transcription in a normal fashion, which is essential for the regulation of biological processes in the normal cervix-uterine environment including vascularization of cervix-uteri after menstruation and normal biochemical processes which are essential for the generation of energy by cervix epithelial cells (Fig. 5b).

## Conclusion

Genotype analysis of PRKCE non-synonymous variants was performed which indicated that the altered genotype-AA of variant E14K with P-value 0.0001, and altered genotype-CC of variant D39H with P-value 0.045, were associated significantly with the risk of the development of cervical cancer. The wet lab analysis also revealed that the variant genotype-AA of E14K is associated with the risk of advanced stage cervical cancer with a P-value of 0.05 and Odds ratio of 7.538. The *In silico* molecular docking and interaction dynamics analysis of wildtype and variants PKCε suggested the potential role of variants in altered structural stability as well as protein-protein interactions that can have oncogenic implications and can serve as potential biomarkers for cancer. Therefore, these variants hold the potential to be further explored via in vitro and in vivo experimentations. Lastly, functional studies of these variants on large cohort-size should be performed to attain in-depth information regarding the molecular functions of these, so that these variants could be used as new therapeutic targets for the treatment of cervical cancer as well as novel biomarkers for better prognosis and early diagnosis of cervical cancer.

## Data Availability

All the used and/or analyzed data during the current study are available from the corresponding author upon reasonable request.
