# Correction: Exploring the prognostic significance of PKCε variants in cervical cancer

**DOI:** 10.1186/s12885-023-11416-x

**Published:** 2023-10-11

**Authors:** Sameen Zafar, Khushbukhat Khan, Yasmin Badshah, Kanza Shahid, Janeen H. Trembley, Amna Hafeez, Naeem Mahmood Ashraf, Hamid Arslan, Maria Shabbir, Tayyaba Afsar, Ali Almajwal, Suhail Razak

**Affiliations:** 1 Department of Healthcare Biotechnology, Atta-Ur-Rahman School of Applied Biosciences, National University of Sciences and Technology, Islamabad, Pakistan; 2 Department of Laboratory Medicine and Pathology, University of Minnesota, Minneapolis, MN USA; 3 Masonic Cancer Center, University of Minnesota, Minneapolis, MN USA; 4 Minneapolis VA Health Care System Research Service, Minneapolis, MN USA; 5 School of Biochemistry and Biotechnology, University of the Punjab, Lahore, Pakistan; 6 University of Bonn, LIMES Institute (AG-Netea), Carl-Troll-Str. 31, 53115 Bonn, Germany; 7 Department of Community Health Sciences, College of Applied Medical Sciences, King Saud University, Riyadh, Saudi Arabia

**BMC Cancer (2023) 23:819**

10.1186/s12885-023-11236-z.

After publication of this article [1], the authors reported that in this article the author name ‘Suhail Razak’ was incorrectly written as ‘F. Suhail Razak’.

The original article [1] has been corrected.

## References

[1] Zafar S, Khan K, Badshah Y (2023). Exploring the prognostic significance of PKCε variants in cervical cancer. BMC Cancer.

